# Estimating outcomes in newborn infants using fuzzy logic

**DOI:** 10.1590/0103-058220143228413

**Published:** 2014-06

**Authors:** Luciano Eustáquio Chaves, Luiz Fernando C. Nascimento

**Affiliations:** 1 Unesp, Guaratinguetá, SP, Brasil; 2 Universidade de Taubaté, Taubaté, SP, Brasil

**Keywords:** models, theoretical, fuzzy logic, infant, newborn, risk factors

## Abstract

**OBJECTIVE:**

To build a linguistic model using the properties of fuzzy logic to estimate the risk of death of neonates admitted to a Neonatal Intensive Care Unit.

**METHODS:**

Computational model using fuzzy logic. The input variables of the model were birth weight, gestational age, 5^th^-minute Apgar score and inspired fraction of oxygen in newborn infants admitted to a Neonatal Intensive Care Unit of Taubaté, Southeast Brazil. The output variable was the risk of death, estimated as a percentage. Three membership functions related to birth weight, gestational age and 5^th^-minute Apgar score were built, as well as two functions related to the inspired fraction of oxygen; the risk presented five membership functions. The model was developed using the Mandani inference by means of Matlab^(r)^ software. The model values were compared with those provided by experts and their performance was estimated by ROC curve.

**RESULTS:**

100 newborns were included, and eight of them died. The model estimated an average possibility of death of 49.7±29.3%, and the possibility of hospital discharge was 24±17.5%. These values are different when compared by Student's t-test (*p*<0.001). The correlation test revealed r=0.80 and the performance of the model was 81.9%.

**CONCLUSIONS:**

This predictive, non-invasive and low cost model showed a good accuracy and can be applied in neonatal care, given the easiness of its use.

## Abstract (es)

**OBJETIVO:**

Construir un modelo lingüístico utilizándose de las propiedades de la lógica *fuzzy* para estimar el riesgo de óbito de recién nacidos internados en Unidad de Terapia Intensiva Neonatal (UTIN).

**MÉTODOS:**

Modelo computacional que se utiliza de la lógica *fuzzy*. Las variables de entrada del modelo fueron peso al nacer, edad gestacional, Apgar de 5º minuto y fracción inspirada de oxígeno de recién nacidos internados en una UTIN privada de Taubaté, São Paulo (Brasil). La variable de salida fue riesgo de óbito, estimado en porcentaje. Se construyeron tres funciones de pertinencia para peso al nacer, edad gestacional y Apgar de 5º minuto y dos para fracción inspirada de oxígeno; el riesgo presentó cinco funciones de pertinencia. En el modelo se utilizó el método de inferencia de Mandani por el programa Matlab^(r)^. Los valores del modelo fueron comparados con los suministrados por especialistas y su desempeño fue estimado por la curva ROC.

**RESULTADOS:**

Se incluyeron a 100 recién nacidos y ocurrieron ocho óbitos. Para el óbito, la posibilidad mediana fue de 49,7±29,3% y, para alta hospitalaria, de 24±17,5%. Esos valores son distintos cuando comparados por la prueba *t* de Student (*p*<0,001). La correlación fue r=0,80 y el desempeño del modelo fue de 81,9%.

**CONCLUSIONES:**

Ese modelo predictivo, no invasivo y de bajo costo financiero mostró buena precisión y se puede usarlo en unidades neonatales, dada su facilidad de aplicación.

## Introduction

The fuzzy logic theory was introduced in 1964 by Zadeh, when he worked with problems of classification of sets that did not have well-defined boundaries. The expression "fuzzy" means cloudy, diffuse, imprecise, and refers to the fact that, in many cases, the systems and set boundaries under analysis are not fully known. There are numerous situations in which the membership relation is not well defined and in such cases, one cannot say exactly whether the element belongs to a given set^(^ 1 ^)^.

The characteristics and the ability to deal with linguistic terms could explain the increase in the number of studies that apply fuzzy logic in Biomedical problems. Indeed, the theory of fuzzy sets became an important mathematical approach in diagnostic systems, medical imaging treatments, and more recently, in epidemiology and in Public Health^(^ 2 ^)^. The application of this theory in the medical field has shown great ability to enhance and develop equipment and models in various hospitals and research activities^(^ 2 ^)^. Thus, a newborn with birth weight of 2,490g and another with 2,510g, classically categorized as low weight and normal birth weight, respectively, and that show no important differences in the biological, anatomical, and physiological aspects, will belong to the low weight and normal weight groups in the fuzzy approach, but with different degrees of membership. The first newborn would have an hypothetical membership of 0.85 in the Low Weight set, and of 0.15 in the Normal Weight set, while the second newborn would have a membership degree of 0.15 in the Low Weight set and of 0.85 in the Normal weight set^(^ 2 ^)^.

Efforts have been concentrated to identify the severity of neonatal diseases, with the creation of systems to predict neonatal morbidity and mortality, also known as neonatal risk scoring systems: Score for Neonatal Acute Physiology (SNAP)^(^ 3 ^)^, Score for Neonatal Acute Physiology - Perinatal Extension (SNAP-PE)^(^ 3 ^)^, Clinical Risk for Babies (CRIB)^(^ 4 ^)^ *, *SNAP II, and SNAP-PE II^(^ 5 ^)^. Although these systems are very useful, some use laboratory tests, making them invasive and an extra risk factor for morbidity and critically ill newborns.

The Neonatal Intensive Care Unit (NICU) is the place that concentrates key human and material resources necessary to provide uninterrupted support to the vital functions of hospitalized newborns. The risk of death for these newborns, especially preterm infants, can be very high, making them a particular group for the study of performance evaluation of the NICU. In this context, the aim of this work was to build a linguistic model using the properties of fuzzy logic, which treats variables not dichotomously - yes or no - but with a degree of uncertainty, in which four input variables (birth weight, gestational age, Apgar score, and fraction of inspired oxygen) and one output variable (risk of death) were defined.

## Method

This is a theoretical model implemented in computer environment, using a fuzzy linguistic model to assess the risk of neonatal death in the NICU. This model is based on the *fuzzification* of the variables: birth weight (BW), gestational age (GA), Apgar score at 5 minutes, and Fraction of inspired oxygen (FiO_2_).

All newborns admitted to the NICU of a tertiary private hospital in Taubaté, SP, from January 1st, 2005 to December 31st, 2006, who had the following data recorded in their medical records: birth weight, gestational age, Apgar score at minutes, and FiO_2_. We excluded those who did not have one or more variables. These data were obtained in the first hours after admission to the NICU. Neonates with malformations of great complexity were excluded. Regarding gestational age and birth weight at the lower limit, the model included the minimum values of 22 weeks and 500g. Thus, patients admitted with gestational age lower than 22 weeks or weighing less than 500g, with very little chances of survival, were not analyzed because the model itself rejected them.

**Figure 1:**
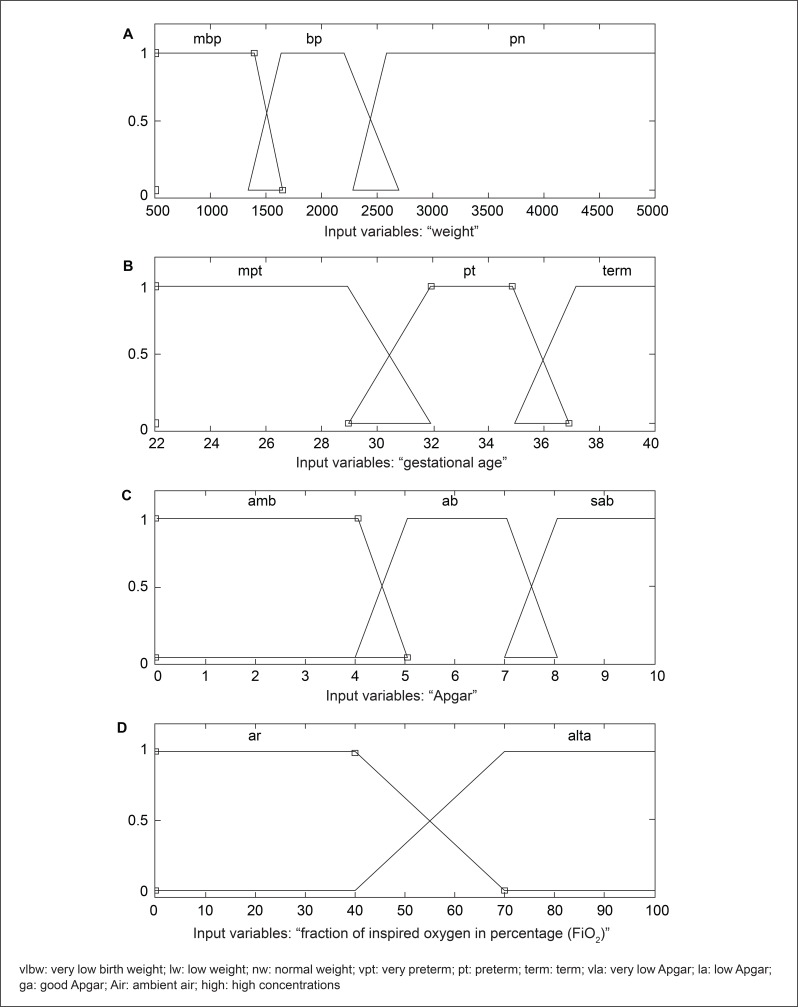
Input variables. (A) birth weight in grams (weight); (B) gestational age in weeks (ga); (C) Apgar score; (D) fraction of inspired oxygen in percentage (FiO2). Taubaté, 2005-2006

**Figure 2:**
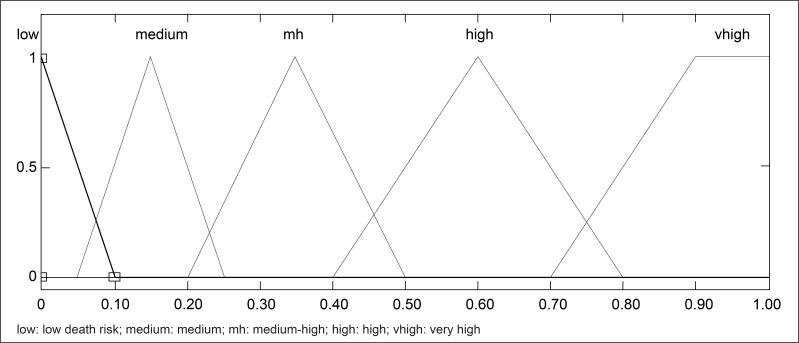
Output variable: neonatal death risk. Taubaté, 2005-2006

The model was developed with the help of an expert who produced three fuzzy sets for the variable birth weight: very low birth weight, low birth weight, and normal birth weight; three fuzzy sets for the variable GA: very preterm, preterm, and term; three fuzzy sets for the Apgar score: low, medium, and high; and two fuzzy sets for the variable FiO_2_: low and high (Figure 1). The output is the risk of death with the five variables: very high, high, medium high, medium, and low (Figure 2). It is important to note that, unlike sets commonly studied, i.e., with definite boundaries such as "low weight is up to 2,499g", on the fuzzy approach these limits are not precise. On the other hand, the cutoff values for the classes of birth weight, gestational age, and Apgar score were respected, corresponding to the intersections of the lines of relevance functions (Figure 2).

In combining all possible inputs, 54 rules were drawn up; of these, 14 were excluded, leaving 40 on the model. The excluded rules showed no plausibility to clinical situations, such as, for instance: neonate with gestational age very preterm, with normal birth weight, Apgar score lower than 7, and low FiO_2_. Another example would be a term newborn, with normal weight, good Apgar and high FiO_2_.

The procedure of the fuzzy linguistic model, given the previous entries for all newborns, is to calculate the degree of membership of these values in all fuzzy sets of birth weight, gestational age, Apgar score, and FiO_2_. Then, the risk of neonatal death is determined by the fuzzy interference method proposed by Mandani, applying the technique of defuzzification of the center of the area in the model's output set. 

To evaluate the performance of the model, 25 cases were offered, part of the set of newborns studied by four specialists in neonatology, different from those who idealized the model. All professionals had over 10 years of experience and were Neonatologists certified by the Brazilian Society of Pediatrics (Sociedade Brasileira de Pediatria - SBP), who had to estimate the risk of death (as percentage) in the NICU. The source of such information was not reported to the consultants to prevent a bias; the information was provided as if they were hypothetical cases so that the risk of death was estimated. Then, the means among these four specialists for the 25 cases were estimated. The situations were analyzed by the constructed model. The values obtained in both cases, models, and specialists corresponded to means of the Pearson correlation, obtaining the correlation coefficient (r) and statistical significance.

Data for the variables birth weight, gestational age, Apgar score, and FiO_2_ were entered into an Excel spreadsheet that, through a routine of the Matlab^(r) ^program, was imported into the fuzzy model, providing the risks of death for each case. The mean values of the model variables, besides the obtained score, were compared using Student *t *test, establishing significance at *p*<0.05.

The performance of the model, along with its confidence interval of 95%, was estimated by the ROC curve provided by the Statistical Package for the Social Sciences (SPSS), student version.

The study was approved by the Research Ethics Committee of the hospital where it was developed.

## Results

During the study period, 100 patients met the inclusion criteria, with no incomplete information on the medical records. Of the total number of patients, eight died (8%). Mean, standard deviation (SD), minimum and maximum of the variables weight, GA, Apgar score, FiO_2, _and risk of death are shown in Table 1.

**Table 1:**
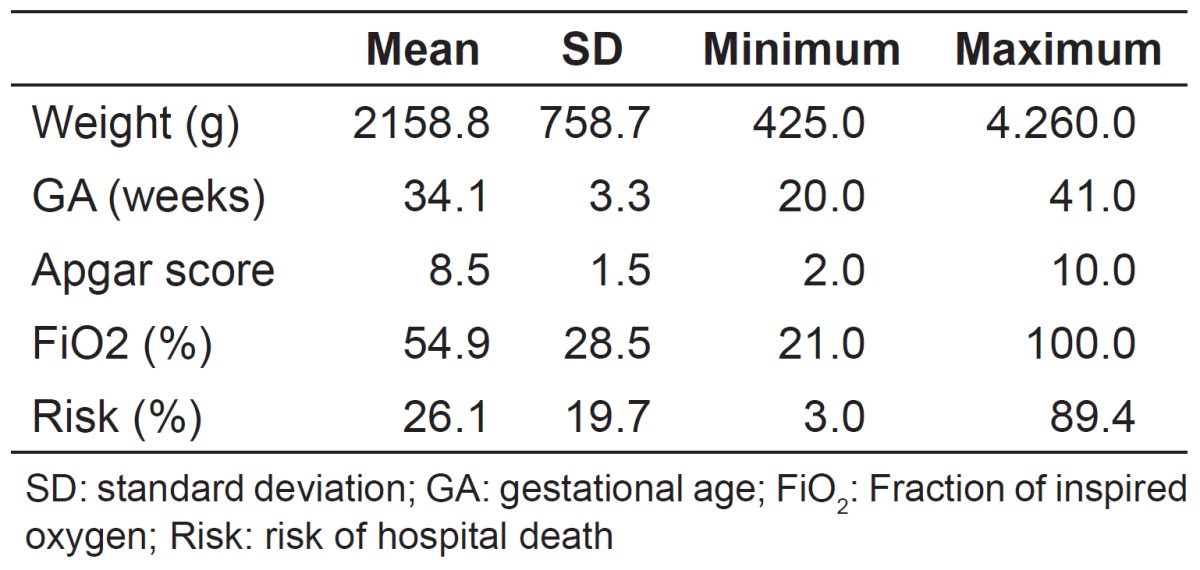
Mean values, standard deviation, minimum and maximum of the variables: birth weight, gestational age, Apgar score, and fraction of inspired oxygen in newborns hospitalized in Intensive Care Unit. Taubaté, 2005-2006

Table 2, presents the mean values of the variables birth weight, GA, Apgar score, FiO_2_, and the risk estimated by the model, regarding hospital discharge and death and their respective *p*-values. A significant difference can be noticed between the risks predicted by the model according to the outcome.

**Table 2:**
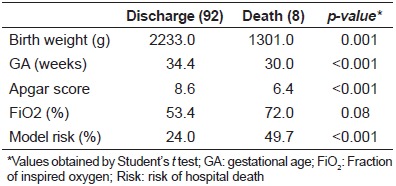
Mean values of the variables: birth weight, gestational age, Apgar score, and fraction of inspired oxygen, according to the king of output - discharge of death. Taubaté, 2005-2006

**Figure 3:**
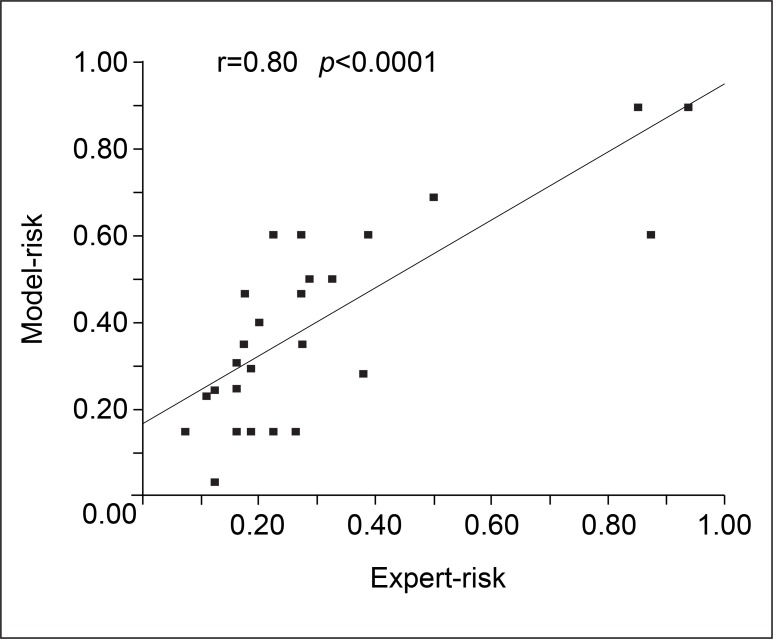
Correlation between the risk of experts and the risk of the model. Taubaté, 2005-2006

The correlation between the mean values given by four experts in the analysis of 25 real cases at the ICU and the values provided by the model are presented in Figure 3, showing a good correlation. When compared to the correlations between risks provided by each of the fours experts and those provided by the proposed model, the values of the Pearson correlation were 0.76; 0.78; 0.80 and 0.76. 

**Figure 4:**
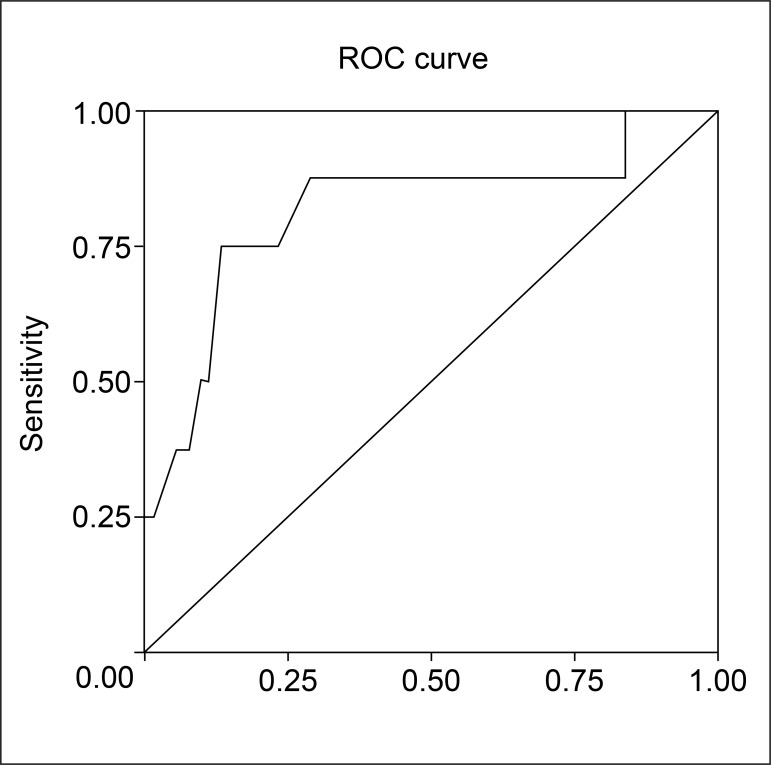
ROC curve for the fuzzy model. Taubaté, 2005-2006

The ROC curve is presented in Figure 4. The performance of the model was of 81.9% (95%CI 63.4-100; *p*=0.003). The wide range is possibly due to the fact that eight deaths were reported (8% of the sample), also explaining the shape of the ROC curve in leaps.

## Discussion

This study deals with the estimation of death at NICUs using the fuzzy logic. The interpretation of mortality rates should be made considering the clinical conditions of newborns, quality of care, availability of resources, and changes in therapeutic approaches used at birth, which differ among countries, cities, hospitals, and neonatal units^(^ 6 ^)^. The fuzzy interference systems are widely used tools for modeling problems of Biomedical engineering, given the nebulous nature of the variables involved. 

Over the past 20 years, physicians working in intensive care have been devoted to develop and validate predictive scores. With this procedure, several goals can be achieved, the main one being the assessment, with a more accurate precision, of the prognosis of a group of patients regarding mortality and morbidity^(^ 7 ^,^ 8 ^)^. 

Two other studies using the fuzzy logic were conducted in Brazil; one of them^(^ 2 ^)^ used two input variables to estimate the risk and the other used data from certificates of live birth^(9) ^to estimate the risk of neonatal death, without considering whether it occurred or not at the NICU. In the first study, there was no validation of the model and, in the second, data were obtained from SINASC and referred to the neonatal mortality in a municipality, considering all the local hospitals, which possibly have different teams of physicians and neonates with a varied clinical profile.

Prematurity, in most studies, is reported as one of the causes that contribute to low birth weight and to Apgar score at 5 minutes lower than 7. The concept of prematurity was based only on the weight, but, from 1970, the World Health Organization (WHO) established that all live births before 37 weeks of gestation were preterm. Among the reasons that lead to preterm birth, we can emphasize those related to maternal, fetal, placental (hemorrhage and placental abruption), and iatrogenic factors, as well as early birth^(^ 10 ^)^. Studies of neonatal mortality have verified that the lower the Apgar score at 5 minutes of life, the lower the chances of survival^(^ 11 ^)^. Low birth weight and prematurity are universally recognized as the most important risk factors for neonatal mortality^(^ 12 ^)^. Thus, as expected, this study found a significant association between neonatal mortality and low birth weight, low gestational weight, Apgar score lower than 7, and Fraction of inspired oxygen greater than 70%.

The major limitation of the existing scores is the fact that their application is very difficult or very complex, causing excessive time demand. These scores use laboratory analyses that are often invasive^(^ 3 ^-^ 5 ^)^. 

This fuzzy model worked with 100 cases for validation. In existing literature, there are few references to studies on the subject in the context of fuzzy logic. In this study, we proposed a fuzzy linguistic model to evaluate the risk of neonatal death based on birth weight, gestational age, Apgar score at 5 minutes, and FiO_2_. The model is easy to apply and it is not invasive, not bringing any risk to the newborn, besides requiring little time for its application. The model presented in this study had satisfactory results when compared to the mean values obtained by experts, and confirmed its predictive ability for the occurrence of hospital death, with an area under the ROC curve of 81.9%. This accuracy value is lower than the accuracy obtained by the CRIB and SNAP-PE scores, which are around 90%^(^ 4 ^,^ 5 ^)^.

The advantage of the predictor of neonatal death risk is that the values of the model do not change over time, even when they are compared separately, that is, in different services; and the same cannot be said for experts' opinions, who can issue them differently, depending on each one's experience. This model avoids variations in the analysis of the newborns' conditions by different professionals, who could use different treatments for the same case^(^ 1 ^)^.

The correlation between the model and the experts is better in extreme situations, where there are fewer uncertainties, e.g., when the values for birth weight, gestational age, Apgar score, and FiO_2_ are either excellent or critical, because the expected result leaves little doubt. On the other hand, when the variables are intermediate, experts have different opinions, which may be the result of individual experiences or may be related to their feelings and expectations. Estimators based on subjective evaluations may vary depending on the conditions of the experts, for instance, if they are under stress, fatigue, and complications in the neonatal unit. 

Some means to identify the risk of neonatal death may provide additional information so that the medical team caring for these infants may get into action and prevent undesirable complications^(^ 9 ^)^. The model presented could offer a standardization of the classification process. 

A possible limitation of this study is in the 2-year time series, resulting in a small number of hospitalizations (100 cases), among which occurred eight deaths, which may have influenced the outcome of the model. The validity of the model should be questioned in public intensive care units and in services in other locations. To validate the model in these units, an additional difficulty would be the consent of these services to provide information on the hospitalized newborns. On the other hand, the model is robust and allows a rapid assessment of the patient shortly after its hospitalization to estimate the risk of death, which may direct the treatment to a determined patient, even prior to obtaining the laboratory results. A possible variability of data does not interfere on the outcome, because the model does not work with data as in a logistic regression. Moreover, the actual data were used to validate the model, unlike the logistic or linear regression, in which it is impossible to build a model without the data.

This study shows a path to be followed in the medical and hospital sector, with the fuzzy approach, which can bring benefits to both the physicians and their patients, contributing to a greater depth of knowledge in the process of diagnosis and treatment, as well as to control medical procedures. An accurate prognosis is critical for improvements in the field of neonatology that uses and refines techniques to save lives. To use a good prognosis index allows the identification of the components of the structure of the unit related to the outcome, as it may, in the future, help the medical team to make ethical decisions and to identify patients and clinical situations in which the benefit of intensive therapy is very low and the cost, very high.

The fuzzy model is very simple and of low financial cost, making it thus possible to be deployed. A model with such a simple structure may be easily converted into a computer program that can even be used on handheld computers. Furthermore, the use of non-invasive measurements, such as those for obtaining the blood biochemical parameters, makes the application of the fuzzy model quite attractive. This model can be an ally of intensive care pediatricians and of pediatricians who work in municipalities where there is no expert available, and could be used as a support in the care of the newborn.
