# Supplementary material for: Estimating outcomes in newborn infants using fuzzy logic
Source: Rev Paul Pediatr. 2014 Jun;32(2):164–70. doi: 10.1590/0103-058220143228413 (PMC4183016; doi:10.1590/0103-058220143228413)
Supplement: Translation

# Translation: Estimando o desfecho no recém-nascido usando lógica *fuzzy*

**Authors:** Luciano Eustáquio Chaves, Luiz Fernando C. Nascimento

**Affiliations:** 1 Unesp, Guaratinguetá, SP, Brasil; 2 Universidade de Taubaté (Unitau), Taubaté, SP, Brasil

**Keywords:** modelos teóricos, lógica *fuzzy*, recém-nascido, fatores de risco

## Abstract

**OBJETIVO:**

Construir um modelo linguístico utilizando-se as propriedades da lógica *fuzzy* para estimar o risco de óbito de recém-nascidos internados em Unidade de Terapia Intensiva Neonatal (UTIN).

**MÉTODOS:**

Modelo computacional utilizando a lógica *fuzzy*. As variáveis de entrada do modelo foram peso ao nascer, idade gestacional, Apgar de 5º minuto e fração inspirada de oxigênio de recém-nascidos internados em uma UTIN privada de Taubaté, SP. A variável de saída foi risco de óbito, estimado em percentagem. Construíram-se três funções de pertinência para peso ao nascer, idade gestacional e Apgar de 5º minuto e duas para fração inspirada de oxigênio; o risco apresentou cinco funções de pertinência. No modelo, utilizou-se o método de inferência de Mandani pelo progama Matlab^(r)^. Os valores do modelo foram comparados com os fornecidos por especialistas e seu desempenho foi estimado pela curva ROC.

**RESULTADOS:**

Incluíram-se 100 recém-nascidos e ocorreram oito óbitos. Para o óbito, a possibilidade média foi de 49,7±29,3% e, para a alta hospitalar, de 24±17,5%. Esses valores são diferentes quando comparados pelo teste *t *de Student (*p*<0,001). A correlação foi r=0,80 e o desempenho do modelo foi de 81,9%.

**CONCLUSÕES:**

Esse modelo preditivo, não invasivo e de baixo custo financeiro mostrou boa acurácia e pode ser usado em unidades neonatais, dada a facilidade de sua aplicação.

## Introdução

A teoria de lógica* fuzzy* foi apresentada em 1964 por Zadeh, quando trabalhava com problemas de classificações de conjuntos que não possuíam fronteiras bem definidas. O termo "*fuzzy*" significa nebuloso, difuso, impreciso, e refere-se ao fato de, em muitos casos, não se conhecerem completamente os sistemas em análise e os limites dos conjuntos. Existem inúmeras situações em que a relação de pertinência não é bem definida e, nesses casos, não se pode dizer com exatidão se o elemento pertence ou não a um dado conjunto^(^ ^1^ ^)^.

As características e a capacidade de lidar com termos linguísticos poderiam explicar o aumento do número de trabalhos que aplicam a lógica *fuzzy* nos problemas de Biomedicina. De fato, a teoria dos conjuntos *fuzzy* tornou-se uma importante abordagem matemática nos sistemas de diagnóstico, no tratamento médico por imagens e, mais recentemente, em epidemiologia e na Saúde Pública^(^ ^2^ ^)^. A aplicação dessa teoria na área médica tem demonstrado grande capacidade para aprimorar e desenvolver equipamentos e modelos nas mais diversas atividades hospitalares e de pesquisa^(^ ^2^ ^)^. Assim, um recém-nascido com peso ao nascer de 2490g e outro com 2510g, categorizados classicamente como baixo peso e peso normal ao nascer, respectivamente, e que não apresentam diferenças importantes nos aspectos biológicos, anatômicos e fisiológicos, na abordagem *fuzzy* pertencerão aos conjuntos de Baixo Peso e Peso Normal, mas com diferentes graus de pertinência. O primeiro recém-nascido teria uma pertinência hipotética de 0,85 no conjunto de Baixo Peso e de 0,15 no conjunto Peso Normal, enquanto o segundo teria um grau de pertinência de 0,15 no conjunto de Baixo Peso e de 0,85 no conjunto Peso Normal^(^ ^2^ ^)^.

Esforços têm sido concentrados para se identificar a gravidade de doenças neonatais, com a criação de sistemas para predizer a morbidade e a mortalidade neonatais, também conhecidos como sistemas de escores de risco neonatal: *Score for Neonatal Acute Physiology *(SNAP)^(^ ^3^ ^)^, *Score for Neonatal Acute Physiology - Perinatal Extension *(SNAP-PE)^(^ ^3^ ^)^,* Clinical Risk for Babies *(CRIB)^(^ ^4^ ^)^ *, *SNAP II e o SNAP-PE II^(^ ^5^ ^)^. Apesar de esses sistemas serem muito úteis, alguns utilizam exames laboratoriais, tornando-os invasivos e mais um fator de risco para morbidade e recém-nascidos criticamente doentes.

A Unidade de Terapia Intensiva Neonatal (UTIN) é o local que concentra os principais recursos, humanos e materiais, necessários para dar suporte ininterrupto às funções vitais dos recém-nascidos internados. O risco de óbito desses recém-nascidos, principalmente prematuros, pode ser muito elevado, tornando-os um grupo particular para o estudo de avaliação de desempenho da UTIN. Nesse contexto, o objetivo deste trabalho foi construir um modelo linguístico utilizando-se as propriedades da lógica *fuzzy*, que trata as variáveis não de forma dicotômica - sim ou não -, mas com um grau de incerteza, no qual se definiram quatro variáveis de entrada (peso ao nascer, idade gestacional, Boletim de Apgar e fração inspirada de oxigênio) e uma variável de saída (risco de morte).

## Método

Trata-se de modelo teórico implementado em ambiente computacional, usando-se modelo linguístico *fuzzy* para avaliar o risco de morte neonatal em UTIN. Esse modelo é baseado na *fuzificação *das variáveis: peso ao nascer (PN), idade gestacional (IG), boletim de Apgar no 5º minuto e a fração inspirada de oxigênio (FiO_2_).

Incluíram-se todos os recém-nascidos admitidos na UTIN de um hospital terciário e privado da cidade de Taubaté, SP, de 1º de janeiro de 2005 a 31 de dezembro de 2006, e que tinham os seguintes dados anotados em seus prontuários: peso ao nascer, idade gestacional, boletim de Apgar no 5º minuto e FiO_2_. Excluíram-se aqueles que não apresentavam uma ou mais variáveis. Obtiveram-se esses dados nas primeiras horas após a internação na UTIN. Excluíram-se os neonatos com malformações de grande complexidade. Quanto à idade gestacional e peso ao nascer no limite inferior, o modelo contemplou os valores mínimos de 22 semanas e 500g. Desta forma, pacientes admitidos com idade gestacional inferior a 22 semanas ou com peso inferior a 500g, considerados com muito pouca probabilidade de sobrevida, não foram analisados porque o próprio modelo os rejeitou.

Desenvolveu-se o modelo com o auxílio de um especialista que elaborou três conjuntos *fuzzy* para a variável peso ao nascer: muito baixo peso, baixo peso e peso normal; três conjuntos *fuzzy* para a variável IG: muito pré-termo, pré-termo e termo; três conjuntos *fuzzy* para o boletim de Apgar: baixo, médio e alto; e dois conjuntos *fuzzy* para a variável FiO_2_: baixa e alta (Figura 1). A saída é o risco de morte com as cinco variáveis: muito alto, alto, médio alto, médio e baixo (Figura 2). É importante notar que, ao contrário de conjuntos habitualmente estudados, isto é, com limites definidos como "baixo peso é até 2499g", na abordagem *fuzzy *esses limites não são precisos. Por outro lado, os valores de corte para as classes de peso ao nascer, idade gestacional e boletim de Apgar foram respeitados, correspondendo aos cruzamentos das retas das funções de pertinência (Figura 2).

**Figura 1:** Variáveis de entrada. (A) peso ao nascer em gramas (peso); (B) idade gestacional em semanas (idg); (C) escore de Apgar; (D) fração inspirada de oxigênio em porcentagem (FiO2). Taubaté, 2005-2006

**Figura 2:** Variável de saída: risco de morte neonatal. Taubaté, 2005-2006

Ao se combinarem todas as possíveis entradas, elaboraram-se 54 regras; destas, 14 foram excluídas, restando, assim, 40 no modelo. As regras excluídas não apresentaram plausibilidade com as situações clínicas, como, por exemplo: neonato com idade gestacional muito pré-termo, com peso ao nascer normal, Apgar inferior a 7 e FiO_2_ baixa. Outro exemplo seria recém-nascido a termo, com peso normal, Apgar bom e FiO_2_ elevada.

O procedimento do modelo linguístico *fuzzy*, dadas as entradas anteriores para todos os recém-nascidos, consiste em calcular o grau de pertinência desses valores em todos os conjuntos *fuzzy* de peso ao nascer, idade gestacional, boletim de Apgar e FiO_2_. Em seguida, o risco da morte neonatal é determinado pelo método de inferência *fuzzy* proposto por Mandani, aplicando a técnica de defuzificação do centro de área no conjunto de saída do modelo (Figura 2). 

Para avaliar o desempenho do modelo, ofereceram-se 25 casos que fazem parte do conjunto dos recém-nascidos estudados para quatro especialistas em Neonatologia, distintos daqueles que idealizaram o modelo. Todos os profissionais apresentavam mais de dez anos de experiência e eram titulados em Neonatologia pela Sociedade Brasileira de Pediatria (SBP), devendo estimar o risco (em porcentagem) de óbito na UTIN. Não se relatou a origem dessas informações aos consultores para não ser criado um viés; as informações foram fornecidas como se tratassem de casos hipotéticos a fim de que fosse estimado o risco de óbito. Em seguida, estimaram-se as médias entre esses quatro especialistas para os 25 casos. Analisaram-se as situações pelo modelo construído. Os valores obtidos em ambas as situações, modelo e especialistas, corresponderam-se por meio da correlação de Pearson, obtendo-se o coeficiente de correlação (r) e a significância estatística.

Os dados das variáveis peso ao nascer, idade gestacional, Boletim de Apgar e FiO_2_ foram inseridos em uma planilha Excel que, por meio de uma rotina do programa Matlab^(r)^, foi importada para o modelo *fuzzy,* fornecendo-se os riscos de óbito para cada caso. Os valores médios das variáveis do modelo, além do escore obtido, foram comparados por meio do teste *t* de Student, sendo significante *p*<0,05.

O desempenho do modelo, juntamente com seu intervalo de confiança de 95%, foi estimado por meio da curva ROC fornecida pelo programa *Statistical Package for the Social Sciences* (SPSS), versão estudante.

O estudo foi aprovado pelo Comitê de Ética em Pesquisa do hospital onde foi desenvolvido. 

## Resultados

Durante o período de estudo, 100 recém-nascidos preencheram os critérios de inclusão, não havendo informação incompleta nos prontuários. Do total, oito foram a óbito (8%). A média, o desvio padrão, o mínimo e o máximo das variáveis peso, IG, boletim de Apgar, FiO_2 _e risco de morte estão apresentados na Tabela 1.

**Tabela 1:** Peso ao nascer, idade gestacional, boletim de Apgar, fração inspirada de oxigênio e risco de óbito hospitalar de recém-nascidos internados em

Na Tabela 2, há os valores médios das variáveis peso ao nascer, IG, boletim de Apgar, FiO_2_ e o risco estimado pelo modelo quanto à alta e ao óbito e os respectivos valores *p*. Nota-se diferença significante entre os riscos preditos pelo modelo segundo o desfecho.

**Tabela 2:** Peso ao nascer, idade gestacional, boletim de Apgar, fração inspirada de oxigênio e risco de óbito hospitalar conforme o tipo de saída - alta ou óbito. Taubaté, 2005-2006

A correlação entre a média dos valores fornecidos por quatro especialistas na análise de 25 casos reais da UTIN e os valores fornecidos pelo modelo está na Figura 3, verificando-se boa correlação. Quando comparadas as correlações entre os riscos fornecidos por cada um dos quatro especialistas e o fornecido pelo modelo proposto, os valores do coeficiente de correlação de Pearson foram 0,76; 0,78; 0,80 e 0,76. 

**Figura 3:** Correlação entre o risco dos especialistas e o modelo. Taubaté, 2005-2006

A curva ROC é apresentada na Figura 4. O desempenho do modelo foi de 81,9% (IC95% 63,4--100; *p*=0,003). O intervalo grande possivelmente se deve ao fato de terem sido relatados oito óbitos (8% da amostra), explicando também o formato da curva ROC em saltos.

**Figura 4:** Curva ROC para o modelo fuzzy. Taubaté, 2005-2006

## Discussão

Este estudo trata da estimação de óbito em UTIN utilizando-se a lógica *fuzzy*. A interpretação dos índices de mortalidade deve ser feita considerando-se as condições clínicas dos recém-nascidos, a qualidade dos cuidados prestados, a disponibilidade de recursos e as modificações dos enfoques terapêuticos usados no momento do nascimento e que diferem entre países, cidades, hospitais e unidades neonatais^(^ ^6^ ^)^. Os sistemas de inferência *fuzzy *são ferramentas muito utilizadas para a modelagem de problemas de Engenharia Biomédica, dada a natureza nebulosa das variáveis normalmente envolvidas. 

Nos últimos 20 anos, os médicos que atuam em terapia intensiva têm se dedicado a desenvolver e validar escores preditivos. Com esse procedimento, vários objetivos podem ser atingidos, sendo o principal a avaliação, com precisão mais acurada, do prognóstico de um grupo de pacientes quanto à mortalidade e à morbidade^(^ ^7^ ^,^ ^8^ ^)^. 

Dois outros estudos foram desenvolvidos no Brasil utilizando-se a lógica *fuzzy*, sendo que um^(^ ^2^ ^)^ usou duas variáveis como entrada para estimar o risco e o outro usou dados da Declaração de Nascido Vivo^(9) ^para estimar o risco de óbito neonatal, sem considerar se o mesmo ocorreu ou não em UTIN. No primeiro estudo, não houve validação do modelo e, no segundo, os dados foram obtidos do SINASC e se referiam à mortalidade neonatal de um município, considerando-se todos os hospitais locais, que contam possivelmente com equipes de médicos diferentes e com neonatos de perfil clínico variado.

 A prematuridade, na maioria dos estudos, é relatada como uma das causas que contribuem para o baixo peso ao nascer e para o Apgar no 5º minuto de vida menor que 7. O conceito de prematuridade baseava-se apenas no peso, mas, a partir de 1970, a Organização Mundial da Saúde (OMS) estabeleceu como prematuro todo nascido vivo antes da 37ª semana de gestação. Dentre as razões que levam ao parto prematuro, destacam-se aquelas relacionadas aos fatores maternos, fetais, placentários (hemorragias e descolamento prematuro da placenta), iatrogênicos e antecipação do parto^(^ ^10^ ^)^. Os estudos de mortalidade neonatal têm verificado que, quanto menor o boletim de Apgar no 5º minuto de vida, menor é também a chance de sobrevivência^(^ ^11^ ^)^. Baixo peso ao nascer e prematuridade são reconhecidos universalmente como os fatores de risco mais importantes para mortalidade neonatal^(^ ^12^ ^)^. Assim, como já esperado, neste estudo encontrou-se associação significativa entre mortalidade neonatal e baixo peso ao nascer, idade gestacional baixa, boletim de Apgar menor que sete e fração de oxigênio maior que 70%.

A grande limitação dos escores que já existem é o fato de sua realização ser difícil ou muito elaborada, ocasionando excessiva demanda de tempo. Esses escores utilizam análises laboratoriais que costumam ser invasivas^(^ ^3^ ^-^ ^5^ ^)^. 

Este modelo *fuzzy* trabalhou com 100 casos para validação. Na literatura existente, há poucas referências de estudos sobre o assunto no contexto da lógica *fuzzy*. Neste estudo, propôs-se um modelo linguístico *fuzzy* para avaliar o risco de morte neonatal com base em peso ao nascer, idade gestacional, boletim de Apgar no 5º minuto e FiO_2_. O modelo é de fácil aplicação e não invasivo, não trazendo nenhum risco ao recém-nascido, além de pouco tempo para ser aplicado. O modelo aqui apresentado mostrou resultados satisfatórios, quando comparado com os valores médios obtidos pelos especialistas, e confirmou sua capacidade preditiva para a ocorrência de óbito hospitalar, com área sob a curva ROC de 81,9%. Esse valor de acurácia é inferior aos obtidos pelos escores CRIB e SNAP-PE, que estão ao redor de 90%^(^ ^4^ ^,^ ^5^ ^)^.

A vantagem do preditor de risco de morte neonatal é que os valores do modelo não mudam com o passar do tempo, mesmo quando são comparados separadamente, isto é, em diferentes serviços; o mesmo não pode ser dito para as opiniões dos especialistas, que podem emiti-las de modo diferente, dependendo da experiência de cada um. Esse modelo evita as variações na análise das condições dos recém-nascidos por profissionais de saúde diferentes, que poderiam utilizar tratamentos diversos para um mesmo caso^(^ ^1^ ^)^.

A correlação entre o modelo e os especialistas é melhor em situações extremas, nas quais existem menos incertezas, por exemplo nos casos em que o peso ao nascer, a idade gestacional, o boletim de Apgar e a FiO_2_ estão ótimos ou quando essas variáveis estão críticas, pois o resultado esperado deixa poucas dúvidas. Por outro lado, quando as variáveis são intermediárias, os especialistas têm opiniões diferentes, o que pode ser resultado das experiências de cada um ou se relacionar aos seus sentimentos e expectativas. Estimadores baseados em avaliações subjetivas podem variar a depender das condições em que se encontram os especialistas, por exemplo, se estão sob estresse, cansaço e intercorrências na unidade neonatal. 

Alguns meios para identificar o risco de morte neonatal podem oferecer informações adicionais para que a equipe médica que cuida desses recém-nascidos possa entrar em ação e prevenir complicações não desejáveis^(^ ^9^ ^)^. O modelo apresentado poderia oferecer uma padronização do processo de classificação. 

Uma possível limitação deste estudo está na série temporal de dois anos, resultando em um número pequeno de internações (100 casos), dentre as quais ocorreram oito óbitos, o que pode ter influenciado o resultado do modelo. Deve-se questionar a validade do modelo em unidades públicas de terapia intensiva e em serviços de outras localidades. Para validar o modelo nessas unidades, uma dificuldade adicional seria o consentimento desses serviços para fornecer informações dos neonatos internados. Por outro lado, o modelo apresenta uma robustez e permite a rápida avaliação do paciente logo após sua internação para estimar o risco de óbito, podendo direcionar o atendimento para determinado paciente, mesmo antes da obtenção de resultados laboratoriais. Uma possível variabilidade dos dados não interfere no resultado, pois o modelo não trabalha com os dados como em uma regressão logística. Ademais, os dados reais serviram para validar o modelo, diferentemente da regressão logística ou linear, nas quais é impossível construir um modelo sem os dados.

Este estudo mostra um caminho a ser seguido na área médica e hospitalar, com abordagem *fuzzy*, que pode trazer benefícios tanto para os médicos quanto para os pacientes, contribuindo para um maior aprofundamento do conhecimento no processo de diagnóstico e tratamento, bem como para controle de processos médicos. Um prognóstico acurado é crítico para os melhoramentos na área da Neonatologia, que utiliza e aprimora técnicas de salvar vidas. Utilizar um bom índice prognóstico permite identificar os componentes da estrutura da unidade relacionada ao desfecho, assim como poderá, no futuro, ajudar a equipe médica a tomar decisões éticas e a identificar pacientes e situações clínicas nas quais o benefício da terapia intensiva é muito baixo e o custo, muito alto.

O modelo *fuzzy* é muito simples e de baixo custo financeiro, tornando-o, assim, possível de ser implantado. Um modelo com uma estrutura simples como essa é facilmente convertido em um programa computacional, que pode ser usado até mesmo em computadores de mão. Além disso, a não utilização de medidas invasivas, como aquelas para se obterem parâmetros bioquímicos do sangue, torna a aplicação do modelo *fuzzy* bastante atraente. Tal modelo pode constituir-se em um aliado dos médicos pediatras intensivistas ou dos pediatras que trabalham nas cidades onde não há um especialista disponível, podendo vir a ser usado como apoio ao cuidado do recém-nascido.
